# Social Isolation During Adolescence Strengthens Retention of Fear Memories and Facilitates Induction of Late-Phase Long-Term Potentiation

**DOI:** 10.1007/s12035-014-8917-0

**Published:** 2014-10-28

**Authors:** Ji-Hong Liu, Qiang-Long You, Mei-Dan Wei, Qian Wang, Zheng-Yi Luo, Song Lin, Lang Huang, Shu-JI Li, Xiao-Wen Li, Tian-Ming Gao

**Affiliations:** 1 State Key Laboratory of Organ Failure Research, Department of Neurobiology, School of Basic Medical Sciences, Southern Medical University, Guangzhou, 510515 China; 2 Key Laboratory of Psychiatric Disorders of Guangdong Province, Southern Medical University, Guangzhou, 510515 China; 3 Department of Pharmacy, The Third Affiliated Hospital of Southern Medical University, Guangzhou, 510630 China

**Keywords:** Social isolation, Fear memory, Long-term potentiation, BDNF, TrkB receptor

## Abstract

Social isolation during the vulnerable period of adolescence produces emotional dysregulation that often manifests as abnormal behavior in adulthood. The enduring consequence of isolation might be caused by a weakened ability to forget unpleasant memories. However, it remains unclear whether isolation affects unpleasant memories. To address this, we used a model of associative learning to induce the fear memories and evaluated the influence of isolation mice during adolescence on the subsequent retention of fear memories and its underlying cellular mechanisms. Following adolescent social isolation, we found that mice decreased their social interaction time and had an increase in anxiety-related behavior. Interestingly, when we assessed memory retention, we found that isolated mice were unable to forget aversive memories when tested 4 weeks after the original event. Consistent with this, we observed that a single train of high-frequency stimulation (HFS) enabled a late-phase long-term potentiation (L-LTP) in the hippocampal CA1 region of isolated mice, whereas only an early-phase LTP was observed with the same stimulation in the control mice. Social isolation during adolescence also increased brain-derived neurotrophic factor (BDNF) expression in the hippocampus, and application of a tropomyosin-related kinase B (TrkB) receptor inhibitor ameliorated the facilitated L-LTP seen after isolation. Together, our results suggest that adolescent isolation may result in mental disorders during adulthood and that this may stem from an inability to forget the unpleasant memories via BDNF-mediated synaptic plasticity. These findings may give us a new strategy to prevent mental disorders caused by persistent unpleasant memories.

**Electronic supplementary material:**

The online version of this article (doi:10.1007/s12035-014-8917-0) contains supplementary material, which is available to authorized users.

## Introduction

The management of fear memories is important when treating mental health disorders such as posttraumatic stress [1, 2]. In fact, fear memories can also be problematic for individuals who do not meet the diagnostic criteria for a mental health disorder. The rate at which unpleasant memories diminish over time shows interindividual differences: For some, these memories diminish quickly, while others can continue to be affected by these memories for years or even decades [3]. It is therefore crucial to understand the mechanisms underlying these differences since the retention of disturbing memories can significantly disrupt a person’s mental, physical, and social well-being. A behavioral paradigm appropriate for investigating fear memory is Pavlovian fear conditioning. In this paradigm, organisms learn to predict aversive events [4]. In this form of learning, an aversive stimulus (e.g., an electrical shock, loud noise, or unpleasant odor) is associated with a neutral context (e.g., a room) or stimulus (e.g., a tone), resulting in the expression of fear responses to the originally neutral stimulus or context.

Adolescence is a transitional stage of physical and psychological human development that generally occurs from puberty to legal adulthood [5, 6]. During this time, an individual’s social relationships are fundamental to emotional fulfillment, behavioral adjustment, and cognitive function. Previous studies have consistently demonstrated that social isolation predicts morbidity and mortality from cancer, cardiovascular disease, and a host of other diseases [7]. Moreover, social isolation and neglect during adolescence can influence adult cognitive function and social interactions [8–10]. So far, several studies have investigated that social isolation impaired the spatial memory [11–16].

The ability to alter emotional responses is a critical component of normal adaptive behavior and is often impaired in psychological disorders [17]. For example, some forms of anxiety disorders are thought to involve dysfunction in the neural systems underlying the extinction of fear memory [18]. Therefore, we hypothesized that social isolation during adolescence could lead to disruptions in mental health and that the underlying cause of these impairments could result from the inability to forget unpleasant memories. In addition, there are reports showing that social isolation during the late adolescence and adulthoods induces the fear memory deficit [19–23]. Even so, isolation during the early adolescence may have a different consequence and may involve different mechanism, because impaired social behaviors are found only in the mouse isolated starting from P21 but not from P35 [8]. Therefore, we set about to investigate whether social isolation during early adolescence influences fear memories and its mechanisms.

Therefore, the aim of this study was to evaluate the implications of adolescent social isolation on fear memory retention and its underlying cellular mechanisms (i.e., late-phase long-term potentiation [L-LTP]) during adulthood. Moreover, since many studies have demonstrated an intimate relationship between brain-derived neurotrophic factor (BDNF) and L-LTP [24–28], we also studied whether BDNF was involved in this phenomenon.

## Materials and Methods

### Subjects

Adult male C57 BL/6 mice were housed in standard laboratory cages (four to five per cage) on a 12-h light/dark cycle (lights on at 8:00 A.M.) in a temperature-controlled room (21–25 °C). Mice were housed with free access to food and water. At postnatal days 21–22, littermate pups were housed either individually or in groups of 3–5 mice per cage. While isolated animals could hear and smell other animals within the housing facility, there was no physical interaction with other mice. Additionally, the number of investigators handling the isolated animals during weekly cage changes was kept to a minimum [29]. Animals were isolated for 4 weeks before the initiation of any experimental procedure. Behavioral testing was performed during the light cycle between 10:00 A.M. and 5:00 P.M. All procedures were conducted in accordance with the Chinese Council on Animal Care Guidelines [30], and efforts were made to minimize animal suffering and to reduce the number of animals used.

### Drugs

Emetine dihydrochloride hydrate (Emetine; Sigma-Aldrich, USA) was dissolved in distilled water. K-252a (Sigma-Aldrich, USA) was dissolved in dimethyl sulfoxide (DMSO), and the concentration of DMSO (Sigma) used for all solutions was less than 0.1 %. All other chemicals were purchased from Sigma-Aldrich. Dose selections for these drugs were based on both pilot and previously published studies [31, 32].

### Open-Field Test

The open-field test was performed in a rectangular chamber (60 × 60 × 40 cm) composed of gray polyvinyl chloride; the center area of which was illuminated by 25-W halogen bulbs (200 cm above the field). Mice were gently placed into the testing chamber for a 5-min recording period, which was monitored by an automated video tracking system. Images of the paths traveled in those 5 min were automatically calculated using the DigBehv animal behavior analysis program.

### Social Interaction Test

This behavioral test was conducted in darkness. Mice were placed in a new area with a small, empty cage at one end. Baseline movement was tracked for 2.5 min and was then recorded for 2.5 min in the presence of a caged aggressor male. The duration each mouse spent in a predefined interaction zone and other measures were obtained using Ethovision XT (Noldus, USA) software. After each trial, the apparatus was cleaned with a solution of 70 % ethanol in water to remove olfactory cues.

### Novelty Suppressed-Feeding Test

After 24 h of food (but not water) deprivation, mice were placed into the testing box. One single pellet of food was placed on a white piece of paper positioned at the center of the testing box (50 × 50 × 20 cm), the floor of which was covered with 2 cm of thick padding. A stop watch was used to measure 5 min, and latency was scored as the time at which mice began biting the food. Immediately after biting, mice were transferred to their home cage for another 5 min, and food intake amount over this time was measured (home-cage food intake).

### Fear Conditioning Test

Mice were first habituated to the behavioral room and were then allowed to freely explore the apparatus (MED-VFC-NIR-M; Med Associates) for 3 min. During training, mice were placed in a conditioning chamber and exposed to tone-foot shock pairings (tone, 30 s, 80 dB; foot shock, 1 s, 0.4 mA) with an interval of 80 s at 24 h after training. Mice were returned to the chamber to evaluate contextual fear learning. Mice were housed isolated during the whole fear conditioning test. Freezing during training and testing was scored using Med Associates Video-Tracking and scoring software.

### Electrophysiological Recordings

This study’s entire protocol is based on previous studies from our laboratory [33]. In brief, mice were decapitated, and transverse hippocampal slices (400 μm) were prepared using a Vibroslice (VT 1000S; Leica) in ice-cold artificial cerebral spinal fluid (ACSF). For field potential recording, the same ACSF was used for sectioning and recording and contained (in mM) 120 NaCl, 2.5 KCl, 1.2 NaH2PO4, 2.0 CaCl2, 2.0 MgSO4, 26 NaHCO3, and 10 glucose. After sectioning, hippocampal slices were incubated for 30 min at 34 °C and then at room temperature (25 ± 1 °C) for an additional 2–8 h. All solutions were saturated with 95 % O_2_/5 % CO_2_ (*v*/*v*).

Slices were placed in a recording chamber, which was superfused (3 mL/min) with ACSF at 32–34 °C. Field excitatory postsynaptic potentials (fEPSPs) were evoked in the CA1 stratum radiatum by stimulating Schaffer collaterals (SC) with a two-concentrical bipolar stimulating electrode (FHC). The evoked responses were recorded in current-clamp mode by the Axon MultiClamp 700B (Molecular Devices) amplifier with ACSF-filled glass pipettes (1–5 MΩ). Test stimuli consisted of monophasic 100-μs pulses of constant currents (with intensity adjusted to produce 25 % of the maximum response) at a frequency of 0.033 Hz. The strength of synaptic transmission was determined by measuring the initial (10–60 % rising phase) slope of fEPSPs. LTP was induced by one or four stimulus trains (with stimulation at test stimulus intensity) delivered at 100 Hz, with each train having 50 pulses, and an intertrain interval of 10 s. The level of LTP was determined at an average of 0–180 min after tetanic stimulation.

### ELISA

Mice were deeply anesthetized and perfused with 40 mL of Ca2^+^/Mg2^+^-free Dulbecco’s phosphate-buffered saline (DPBS). CNS tissues were harvested, and the cortex, striatum, and hippocampus were dissected. Tissues were disrupted by sonication in NP-40 lysis buffer (50 mM Tris pH = 7.4, 150 mM NaCl, 1% NP-40, 0.1 % Triton X-100, and 0.1 % SDS) supplemented with protease inhibitors. The homogenized material was centrifuged at 20,000×*g* for 15 min, and the cleared supernatant was collected. For detection of BDNF, cleared samples were treated with 1 N HCl for 15 min at room temperature, followed by neutralization with 1 N NaOH. Total protein levels in CNS homogenates were determined by bicinchoninic acid (BCA) assay (Pierce). Cytokines were measured with Platinum mouse ELISA kits (eBioscience), and BDNF levels were measured using the Emax ImmunoAssay System ELISA kit (Promega) according to the manufacturer’s instructions.

### Statistical Analyses

The number of experimental animals is indicated by “*n*.” An independent sample *t* test or one-way analysis of variance (ANOVA) followed by the least significant difference (LSD) for post hoc comparisons was used for statistical analysis throughout the study using SPSS software (SPSS, Inc.). All data are expressed as mean ± SEM. The statistical significance level for all tests was set at *p* < 0.05.

## Results

### Social Isolation During Adolescence Alters Adult Social Interaction and Novelty-Suppressed Feeding

To substantiate the effects of social isolation during adolescence on adulthood, C57 mice were socially isolated following weaning or were reared in normal conditions. After 4 weeks of isolation, the following behavioral tests were performed on the experimental mice: open field, social interaction, and the novelty-suppressed feeding (which entailed food deprivation for 24 h prior to start of the examination). To minimize their suffering, only one batch of mice underwent all the behavioral tests. Moreover, mice were allowed to rest for 3–4 days between tests.

**Fig. 1:**
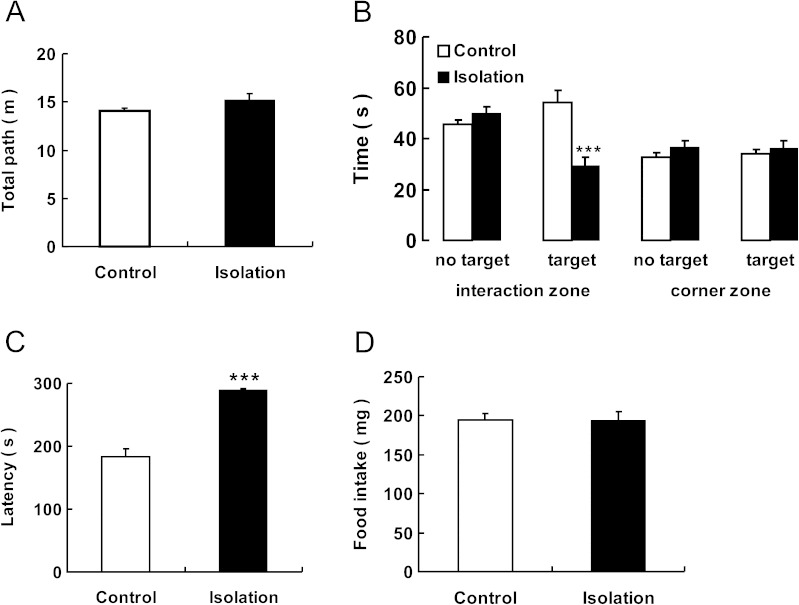
Social isolation during adolescence alters adult social interaction and NSF. **a** Mice tested 4 weeks after isolation from weaning spent the same distance in the open-field test. **b** Isolated mice spent less time in the interaction zone when there was a target mouse than control mice. No difference was observed in the time in the interaction zone when there was no target mouse or corner zone. **c, d** In the novelty-suppressed feeding test, the latency to explore and bite the food was increased, but no difference in food intake amount was observed. *Vertical bars* represent the mean ± SEM. *Asterisks* indicate significant differences from the relevant controls (*n* = 10/group, *p* < 0.05, **p < 0.01, two-tailed *t* test)

Figure 1a demonstrates that social isolation did not affect general locomotor activity as indicated by total path length in the open-field test [*t* (18) = −1.321; *p* = 0.203; Fig. 1a]. However, we found that socially isolated adolescents spent less time interacting with other mice during adulthood when compared to control mice [*t* (18)] = 4.376; *p* < 0.001; Fig. 1b]. Similar results were observed in latency times for the novelty-suppressed feeding test: Social isolation increased the latency to approach and begin eating the food [*t* (18) = −8.452; *p* < 0.001; Fig. 1c] without affecting overall food intake [*t* (18) = 0.067; *p* = 0.948; Fig. 1d]. The above results support the hypothesis that social isolation during adolescence leads to social and mood disorders in adults.

### Adolescent Social Isolation Strengthens the Retention of Fear Memories

**Fig. 2:**
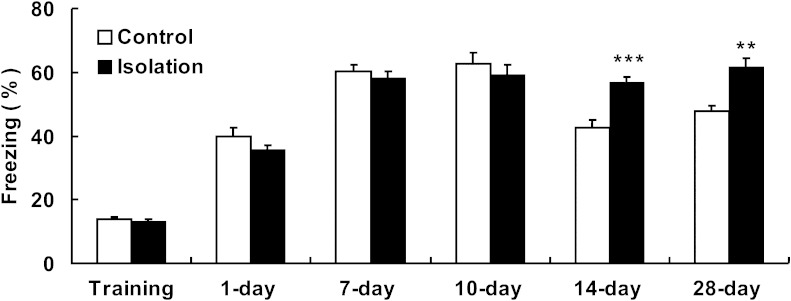
Adolescent social isolation strengthens the retention of fear memories. Fear memories for contextual training 14 and 28 days after training were increased in isolated mice (*n* = 12) compared with control mice (*n* = 10), without any changes in other days. *Vertical bars* represent the mean ± SEM. *Asterisks* indicate significant differences from the relevant controls (***p < 0.001, **p < 0.01, two-tailed)

To examine the effects of the adolescent social isolation on fear memory during adulthood, both control and isolated mice underwent hippocampal-dependent contextual fear conditioning [34]. Briefly, animals learned to fear a neutral conditioned stimulus that had been paired with an aversive unconditioned stimulus (such as foot shock) on the first day. Contextual conditioning was then measured 1, 7, 10, 14, and 28 days after training. We found that mice that had undergone social isolation exhibited a longer retention of fear memory (Fig. 2). Student’s *t* test indicated no significant difference in freezing during training (*t* (20) = 0.702, *p* = 0.491) and context tested at 1 (*t* (20) = 1.343, *p* = 0.199), 7 (*t* (20) = 0.758, *p* = 0.457), and 10 (*t* (20) = 0.747, *p* = 0.464) days between control and isolated mice, but a significant group difference in contextual freezing was found when mice were tested at 14 (*t* (20) = −5.004, *p* < 0.001) and 28 (*t* (20) = −3.746, *p* = 0.001) days. Compared with control mice, isolated mice displayed longer freezing times at the second and fourth weeks after training, indicating strengthened retention of fear memory. In other words, socially isolated adolescents exhibited an inability to forget the intrusive memory of the foot shock in adulthood.

### Social Isolation During Adolescence Facilitates the Induction of Late-Phase Long-Term Potentiation

**Fig. 3:**
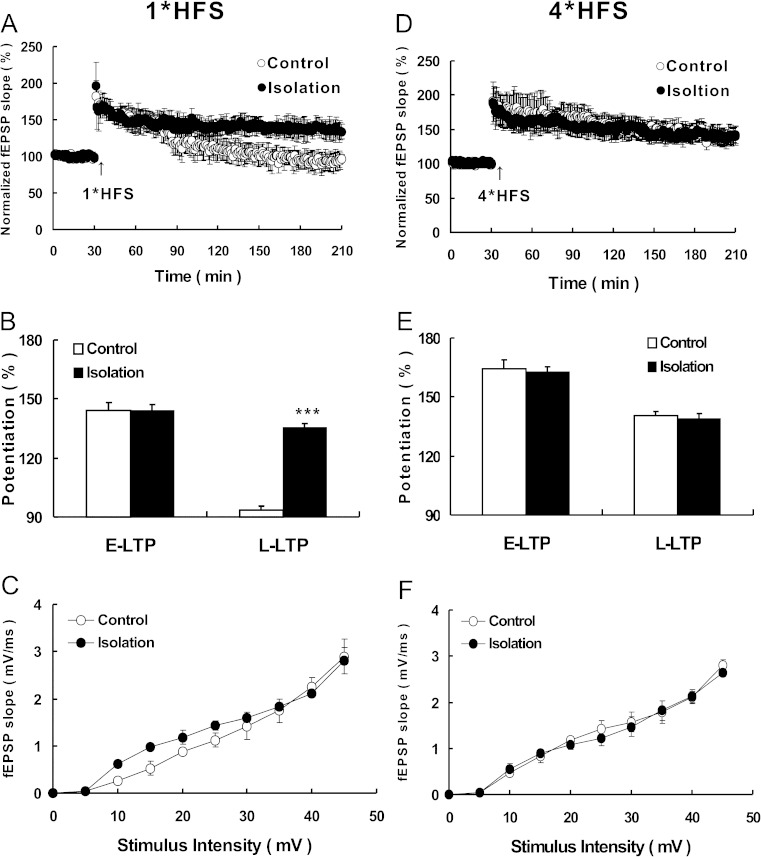
Social isolation during adolescence facilitates the induction of L-LTP. **a, d** Normalized fEPSP slope is plotted from control slices (*open circles*) and isolated slices (*closed circles*). *Arrow* indicates 1*HFS (100 Hz, 1 s) tetanus stimulation in A and 4*HFS (100 Hz, 1 s with 10-s interval) in **d. b, e** Histogram showing average percentage of potentiation after tetanus versus baseline (100 %) at control and isolated mice. *Left* showed the comparing between the E-LTP (40–50 min after tetanus versus baseline) of the two groups; *right* showed the comparing between the L-LTP (170–180 min after tetanus vs baseline). **c, f** Adolescence social isolation does not shift I-O curve. Input/output curve of fEPSP (mV/ms) versus stimulation intensity (mV) were taken from control and isolated mice. *Vertical bars* represent the mean ± SEM. *Asterisks* indicate significant differences from the relevant controls (***p < 0.001, two-tailed)

Hippocampus LTP is assumed to represent the cellular mechanism underlying learning and memory [35, 36]. To investigate the cellular mechanism underlying the social isolation-strengthened fear memory, we recorded fEPSPs in the dendritic region of CA1 and compared the LTP induction between hippocampal slices taken from isolated (isolated for 4 weeks) and control animals. We first induced LTP using one train of high-frequency stimulation (HFS), which was used to induce early-phase LTP (E-LTP) [33, 35, 37–39]. Using this stimulation, we determined that there was no difference in E-LTP between hippocampal slices from isolated and control animals. However, unlike controls, whose L-LTP was absent after 1 h of induction, the L-LTP of slices from isolated mice was maintained for all 3 h (Fig. 3a). In the one-train HFS-induced L-LTP, the slope of fEPSPs was 93.37 ± 1.99 (*n* = 6) and 135.62 ± 2.01 (*n* = 6) in control and isolated groups, respectively [*t* (10) = −47.294; *p* < 0.001; Fig. 3b]. Compared with controls, it was easier to induce L-LTP in slices obtained from isolated animals. We also performed complete input-output (I-O) curves at a series of increasing stimulation intensities in both control and isolated slices and observed no detectable changes in basal synaptic transmission (Fig. 3c).

We then induced LTP using four trains of HFS, which is normally used to induce L-LTP [24, 32, 39]. Using four trains, we found that not only the E-LTP was normal, but also that the L-LTP could be maintained for 3 h in both control and isolated slices (Fig. 3d, e). Moreover, there was no significant difference in I-O curves between control and isolated slices (Fig. 3f), indicating that their basal synaptic transmission was not changed. As L-LTP requires not only the modification of existing proteins and their trafficking at synapses, but also de novo protein synthesis [40–44], we tested whether the four trains of HFS really were induced L-LTP. In order to do this, we employed a commonly used protein synthesis inhibitor emetine (20 μM) and found that L-LTP was impaired in emetine-treated compared to control groups (Fig. S1.A). In the HFS-induced L-LTP, the slope of fEPSPs was 157.01 ± 2.89 and 109.81 ± 3.46 in control and emetine-treated groups, respectively [*t* (8) = 48.538; *p* < 0.001; Fig. S1.B]. Additionally, emetine did not change basal synaptic transmission (Fig. S1.C).

Taken together, our data demonstrated that social isolation during adolescence facilitated the induction of L-LTP in the hippocampus.

### Adolescent Social Isolation Increases BDNF Protein Levels in the Hippocampus

Many genetic and pharmacological studies have suggested that BDNF is necessary for L-LTP [24–28, 45]. Heterozygous BDNF (+/−) knockout mice have a significant deficit in L-LTP [45–47]. Moreover, other studies have reported that BDNF mRNA levels increase in the hippocampal CA1 region and dentate gyrus within 2–4 h after application of L-LTP-inducing tetanic stimulation [48–50]. These investigations collectively revealed the important role of BDNF in the induction of L-LTP. To test whether the facilitated induction of L-LTP in socially isolated mice was due to increased levels of BDNF, we examined BDNF protein levels in control and isolated mice using ELISA.

**Fig. 4:**
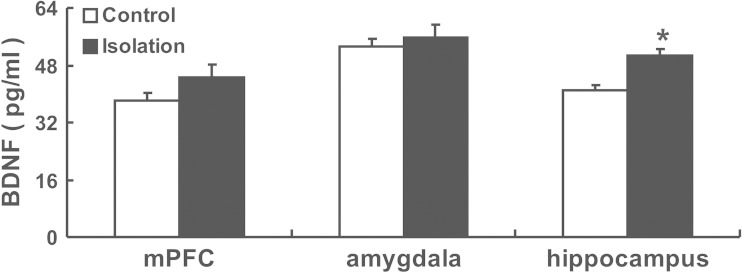
Adolescence social isolation increases the BDNF protein level in hippocampus. Histogram showed BDNF protein level detected by ELISA from mPFC, amygdala, and hippocampus of control and isolated mice. Difference was found only in hippocampus of isolated mice (*n* = 3) compared with the control mice (*n* = 3). *Vertical bars* represent the mean ± SEM. *Asterisks* indicate significant differences from the relevant controls (*p < 0.05, two-tailed)

After 4 weeks of isolation, mice were decapitated, and their brains were quickly removed. We then separated the medial prefrontal cortex (mPFC), amygdala, and hippocampus from control and isolated mice and found significantly increased BDNF protein levels in the hippocampus of socially isolated mice (50.57 ± 1.75 pg/mL) compared with the control mice (40.81 ± 1.58 pg/mL) (*t* (4) = 4.141, *p* = 0.014; Fig. 4). No significant difference was detected in BDNF protein levels in the mPFC (control, 37.96 ± 2.37 pg/mL vs isolation, 44.52 ± 3.78 pg/mL) and amygdala (control, 53.04 ± 2.25 pg/mL vs isolation, 55.75 ± 3.68 pg/mL) between the two groups.

Our results suggest that social isolation during adolescence selectively increases the level of BDNF protein in the hippocampus and that this may contribute to the enhancement of L-LTP induction.

### K-252a Rescues Social Isolation-Facilitated L-LTP

BDNF is a small dimeric protein that works through high-affinity binding with the receptor tyrosine kinase, tropomyosin-related kinase B (TrkB) [51, 52]. BDNF and TrkB are widely distributed across subregions of the adult hippocampus [50]. Previous studies have shown that TrkB [51] and BDNF [52] participated in modulating L-LTP. It has also been shown that K-252a can block L-LTP by inhibiting TrkB receptor [31, 53].

**Fig. 5:**
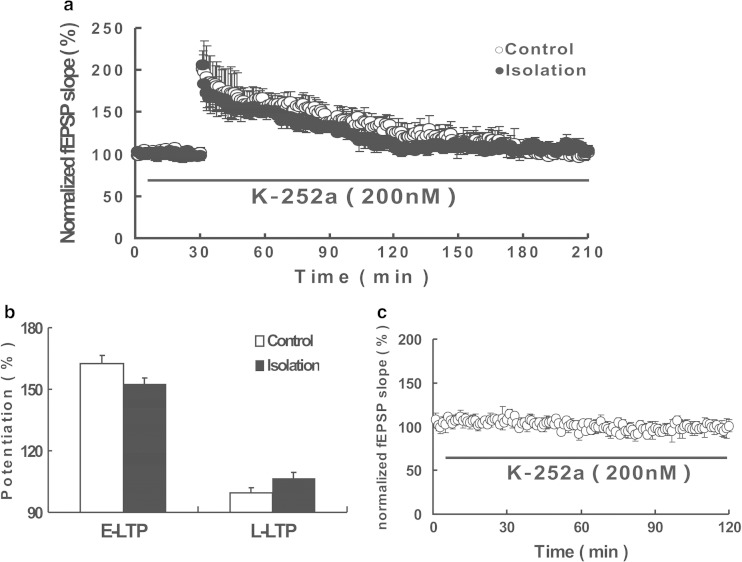
Acutely applied K-252a can rescue the facilitation of L-LTP in isolated mice. **a** Normalized fEPSP slope is plotted from control slices (*open circles*) and isolated slices (*closed circles*), and K-252a (200 nM) was applied starting 20 min before tetanus (*closed circles*). **b** Histogram showed average percentage of potentiation after tetanus versus baseline (100 %) at control and isolated slices. *Left* showed the comparing between the E-LTP (40–50 min after tetanus vs baseline) of the two groups; *right* showed the comparing between the L-LTP (170–180 min after tetanus vs baseline). **c** K-252a did not change the basal synaptic transmission. K-252a was applied 10 min after the beginning. *Vertical bars* represent the mean ± SEM

To further test the involvement of BDNF signaling in facilitated L-LTP, we applied the TrkB receptor inhibitor K-252a and found that a train of HFS could not induce L-LTP in either isolated or control mice (Fig. 5a). The fEPSP slope was 106.35 ± 3.17 in the isolated group, which was similar to that of the control group (Fig. 5b). Moreover, we confirmed that K-252a treatment did not alter the basal synaptic transmission (Fig. 5c).

These data suggested a contribution of the enhanced BDNF signaling in the facilitation of L-LTP caused by social isolation.

## Discussion

The major findings of this study are as follows. First, social isolation during adolescence led to decreased social interaction and increased anxiety-related behavior (as assessed by latency to approach and eat food in the novelty-suppressed feeding test). Second, social isolation strengthened the retention of fear memory and facilitated L-LTP induction in the hippocampus. Third, adolescent social isolation selectively increased hippocampal BDNF protein levels, which facilitated L-LTP via the TrkB receptor. Altogether, our results suggest that social isolation during adolescence disturbs mental health in adulthood, which may result from a disability to forget unpleasant memories via BDNF-mediated synaptic plasticity.

In rodents, it is generally agreed upon that adolescence is the period from weaning to early adulthood and is often marked by discrete developmental stages that have been described by numerous neurobiological and behavioral studies [54]. Clinical and preclinical data implicate that adverse early-life experiences lead to later development of psychiatric and substance abuse disorders [55–57]. Although other covariates should be taken into account, approximately 80 % of young adults who report early-life neglect or abuse are diagnosed with at least one psychiatric illness, including anxiety, affective, schizoaffective, or behavioral disorders [55, 56, 58–62]. To verify this phenomenon, we isolated mice just after weaning and tested the impact of isolation on their behaviors during adulthood, because the age from P21 to P35 is a crucial period for disordered behaviors during adulthood [8]. Our results supported the idea that social isolation during adolescence can cause some mental disorders, and this was reflected in the social interaction and NSF test.

As already mentioned, fear conditioning is an effective behavioral paradigm to arouse unpleasant, intrusive memories. Thus, we next evaluated the retention of fear memories in isolated versus normally housed mice. As expected, isolated mice displayed longer freezing time, indicating that they did not forget the fear of a foot shock when compared with control mice. Our study also showed that there was no obvious difference in the freezing time between control and isolation group until 2 weeks. This is not consistent with other findings. For example, Okada et al. [19] found that social isolation stress impaired consolidation processes of fear memory tested at 24 h after fear conditioning. Besides, fear learning was also disrupted after isolation [20]. These controversies may be interrupted by a different starting time for isolation. In our study, isolation started immediately after weaning whereas a week later in their studies. Those studies indicate that there is remarkable different consequence to the social isolation between the early and late adolescence. In support, isolation starting from P21 but not from P35 can impair social behaviors [8]. LTP is the most widely used paradigm to study cellular and molecular events underlying neuronal plasticity, and it is considered to be a model of learning and memory [36]. LTP can be separated into an early and later phase. E-LTP is short lasting and is independent of new protein synthesis, whereas L-LTP requires activation of cAMP signaling pathway and new protein synthesis [40]. Previous studies have demonstrated that long-term social isolation of adult animals induces a reduction in the size of CA1 and a decrease in synaptic levels of polysialylated neuronal cell adhesion molecule (PSA-NCAM) in the hippocampus [14]. In the current study, we found that social isolation during adolescence can facilitate the induction of L-LTP. As has been shown throughout the literature, L-LTP can only be induced by four (or more) trains of HFS. In our study, however, one train of HFS was adequate to induce L-LTP, implying that isolation during adolescence facilitated the formation of long-term memory. Together, with the results that social isolation during adolescence might lead to certain disruptions in mental health, we hypothesized that isolated mice are unable to forget fear memories that occur during isolation. From these data, we can infer that socially isolated mice might be less capable of dealing with fear memories and that this might predispose them to developing mental disorders. This is in agreement with the fact that symptoms of major depressive disorder (MDD) often overlap with those of depressed moods [63]. It is thus likely that the inability to forget a traumatic memory might cause individuals to exhibit a depressed mood. If this were the case, then this would shed light on new ways to address relieving symptoms of MDD.

In the current study, we also found that socially isolated mice exhibited increased levels of hippocampal BDNF and that the TrkB receptor inhibitor, K-252a, can rescue the facilitation of L-LTP. These findings indicate an involvement of the BDNF/TrkB pathway in the late phase of LTP. One previous study showed that LTP induction evoked increases in BDNF mRNA levels in the CA1 region of the hippocampus [50]. Therefore, our data are consistent with that previous study and reinforce the idea that BDNF is involved in L-LTP. As we all known, biological action of BDNF is mediated by two receptors, the TrkB receptor and p75 [52, 64, 65]. BDNF is first synthesized as a precursor (proBDNF) and is cleaved to form mature BDNF (mBDNF) [66]. Mature BDNF interacts preferentially with TrkB [51], and proBDNF binds p75 with high affinity [67]. TrkB is necessary for L-LTP [45–47], while prior studies indicate that recombinant proBDNF facilitates LTD in hippocampal slices by activation of p75 [65, 68]. As this reason, we selected the TrkB receptor inhibitor to rescue isolation-induced the facilitation of L-LTP. Fear conditioning is dependent on the same brain regions that are highly susceptible to effects of stressors, including the amygdale, hippocampus, and the medial prefrontal cortex [69]. Evidence from lesion studies has shown that patients with hippocampal lesions showed retention deficits [70, 34]. Also, Karl Deisseroth et al. [71] found that hippocampal CA1 optogenetic inhibition blocked remote fear memory recall. And, in our study, we observed a longer retention time and a higher BDNF level in hippocampus in isolation group. Our results give the possibility that hippocampus but not amygdala may be related to the fear memory retention, which should be tested in the future.

In conclusion, these results demonstrate that social isolation during adolescence strengthens the retention of fear memories and facilitates the induction of L-LTP. Moreover, it is conceivable that the inability to forget an unpleasant memory might explain why some individuals develop mental disorders in their adulthood. Taken together, the findings of this study contribute to our current understanding of the negative effects of the unforgotten unpleasant things on the occurrence of mental disorders, which may help to find a new way to relieve them.

## Electronic supplementary material

Below is the link to the electronic supplementary material.

Fig. S1 Emetine impairs the L-LTP. **A**. Normalized fEPSP slope is plotted from control slices (open circles) and emetine treated slices (closed circles) and slices in which emetine (20uM) was applied starting 20 min before tetanus (closed circles). **B**. Histogram showing average percentage of potentiation after tetanus vs baseline (100 %) at control and emetine treated slices. Left showed the comparing between the E-LTP (110-120 min after tetanus versus baseline) of the two groups; Right showed the comparing between the L-LTP (170-180 min after tetanus versus baseline). **C**. Emetine did not change the basal synaptic transmission. Emetine was applied 10 min after the beginning. Vertical bars represent the mean ± the SEM. Asterisks indicate significant differences from the relevant controls (***p < 0.001, two-tailed) (GIF 204 kb)

High resolution image (TIFF 54332 kb)
