# Supplementary figures and images for: Social Isolation During Adolescence Strengthens Retention of Fear Memories and Facilitates Induction of Late-Phase Long-Term Potentiation
Source: Mol Neurobiol. 2014 Oct 28;52(3):1421–9. doi: 10.1007/s12035-014-8917-0 (PMC4588096; doi:10.1007/s12035-014-8917-0)

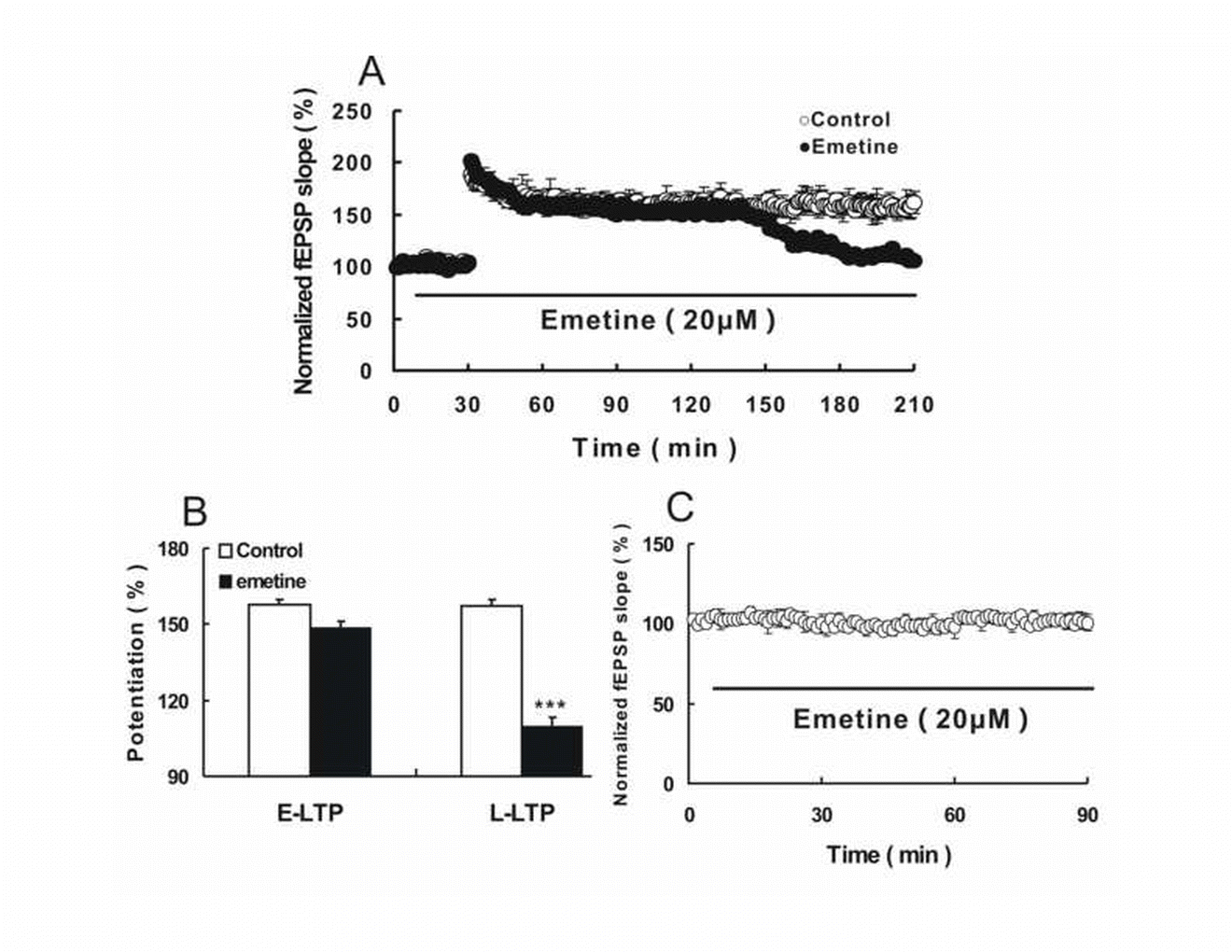

Supplement: Supplementary file 1 — Emetine impairs the L-LTP. A. Normalized fEPSP slope is plotted from control slices (open circles) and emetine treated slices (closed circles) and slices in which emetine (20uM) was applied starting 20 min before tetanus (closed circles). B. Histogram showing average percentage of potentiation after tetanus vs baseline (100 %) at control and emetine treated slices. Left showed the comparing between the E-LTP (110-120 min after tetanus versus baseline) of the two groups; Right showed the comparing between the L-LTP (170-180 min after tetanus versus baseline). C. Emetine did not change the basal synaptic transmission. Emetine was applied 10 min after the beginning. Vertical bars represent the mean ± the SEM. Asterisks indicate significant differences from the relevant controls (***p < 0.001, two-tailed) (GIF 204 kb) [file 12035_2014_8917_Fig6_ESM.gif]
